# Supplementary material for: A high-sensitivity strategy to screen NAD(P)H-dependent reductase activity by coupled enzyme cascade
Source: J Biol Chem. 2026 May 8;302(7):113135. doi: 10.1016/j.jbc.2026.113135 (PMC13264166; doi:10.1016/j.jbc.2026.113135)
Supplement: Supplementary Material [file mmc1.pdf]

Supplementary Information for "A high sensitivity strategy to screen NAD(P)H-dependent reductase activity by coupled enzyme cascade"

Trisha Ghosh[1,†], Jacob Sicheri[1,†], David H. Kwan[1,2,*]

[1]Department of Biology and [2]Department of Chemistry and Biochemistry, Concordia University, 7141 Sherbrooke Street West, Montreal, Quebec, Canada, H4B 1R6

† These authors contributed equally

* To whom correspondence should be addressed

Supplementary Table S1. Z' values and signal-to-noise ratio for LDH assays performed by conventional absorbance assay or fluorescence-based assay by coupled enzyme cascade with different concentrations of NADH

	Absorbance		Fluorescence					
	1 mM NADH				0.1 mM NADH		2 mM NADH	
Concentration of LDH (μg/ml)	Z'	S/N	Z'	S/N	Z'	S/N	Z'	S/N
5	0.82	32	0.83	490	0.80	130	0.33	120
2.5	0.34	16	0.82	480	0.88	150	0.71	130
1.25	-0.41	5.1	0.72	300	0.41	140	0.77	70
0.625	-2.0	2.1	0.75	180	0.86	150	0.72	51
0.313	-14	0.75	0.56	99	0.74	100	0.51	29
0.156	-10	0.76	0.53	56	0.50	66	0.15	18
0.0781	-4.5	1.3	0.43	28	0.60	38	0.25	8.5
0.0391	-38	0.19	0.10	17	0.23	22	0.036	5.0
0.0195	-20	0.35	-1.19	7.0	0.39	9.6	-2.9	1.8
0.00977	-62	0.096	-0.33	5.8	-0.43	7.5	-0.96	2.0
0.00488	-71	0.065	-2.26	2.3	-2.3	3.0	-1.7	1.4
0.00244	-24	0.26	-2.34	2.1	-2.6	1.8	-1.6	1.4

Z' = 1, ideal ; 0.5 < Z' < 1 , excellent ; 0 < Z' < 0.5 , acceptable ; Z' < 0 , not acceptable

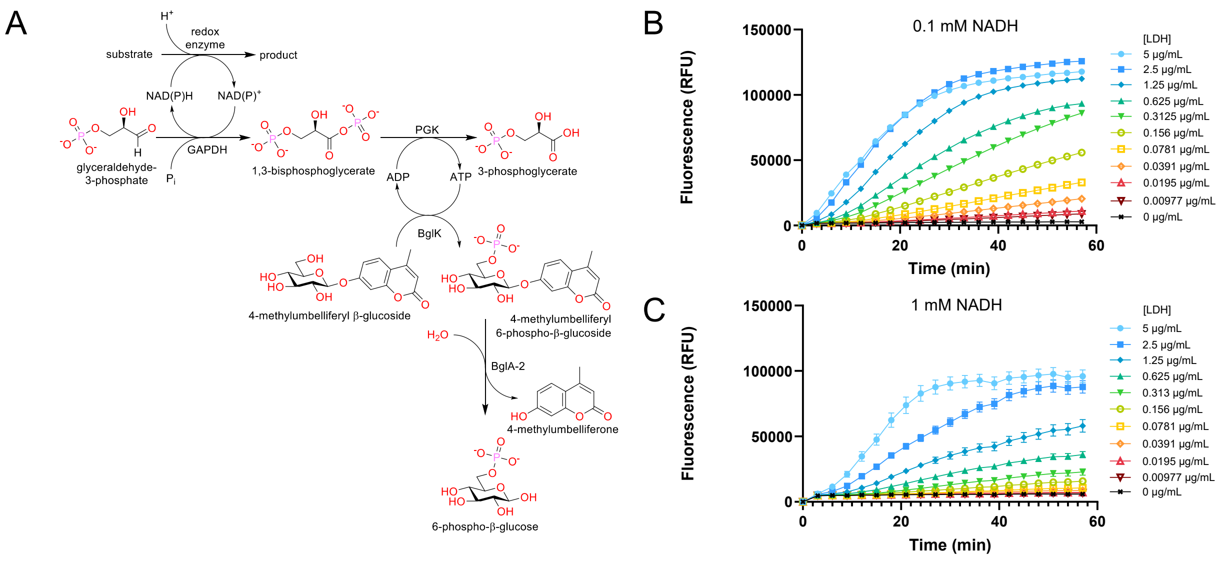

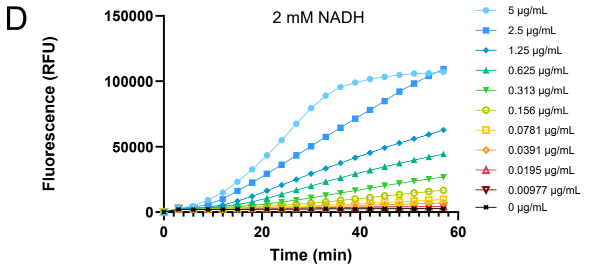

Supplementary Figure S1. Scheme of the standard coupled enzyme cascade (A), with which fluorescence-based assays of the reduction pyruvate catalyzed by LDH (at varied concentration) were performed using NADH included at different concentrations: 0.1 mM NADH (B), 1 mM NADH (C), or 2 mM NADH (D).

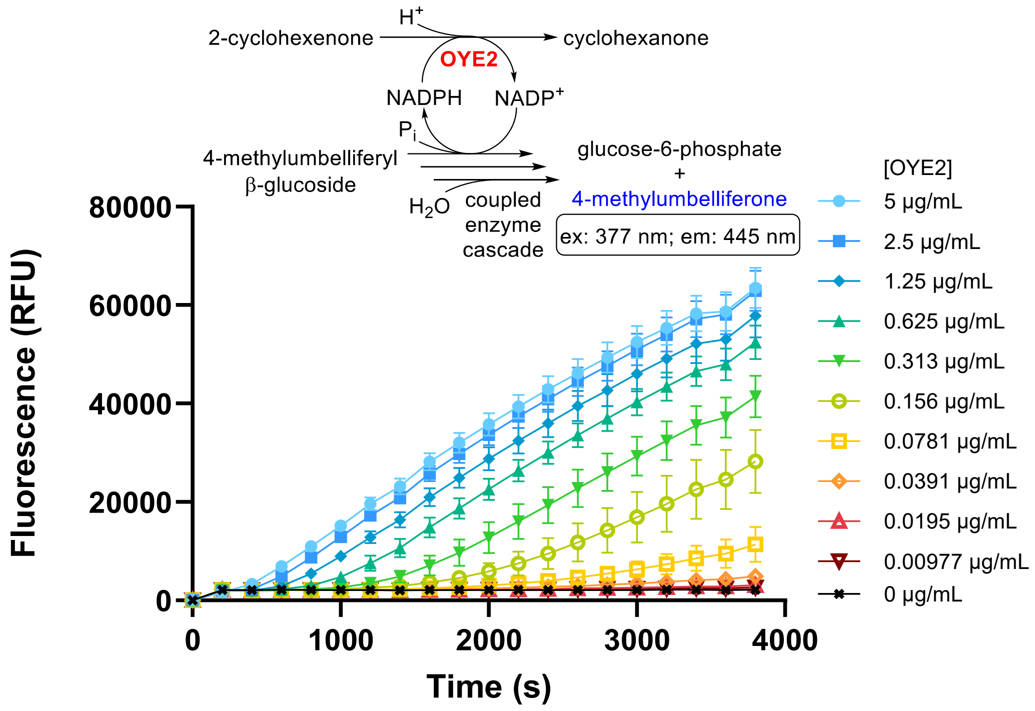

Supplementary Figure S2. Fluorescence-based assay of the reduction of 2-cyclohexenone catalyzed by OYE2 (at varied concentration) with coupled enzyme cascade including coupling enzymes (GapA G188T+P189K, PGK, BglK, & BglA-2), at 20 µg/mL each, along with their (co)substrates (G3P, NADPH, P_i, ADP, MU-Glc).

Supplementary Table S2. Z' values and signal-to-noise ratio for OYE2 assays performed by fluorescence-based assay using a coupled enzyme cascade (including 20 µg/mL each of GapA G188T+P189K, PGK, BglK, and BglA-2)

Concentration of OYE2 (µg/ml)	Z'	S/N
5	0.75	390
2.5	0.82	380
1.25	0.71	330
0.625	0.77	280
0.313	0.57	150
0.156	0.41	47
0.0781	0.13	12
0.0391	-0.35	5.0
0.0195	-1.1	2.8
0.00977	-1.4	2.3
0.00488	-2.7	1.6
0.00244	-2.3	1.8

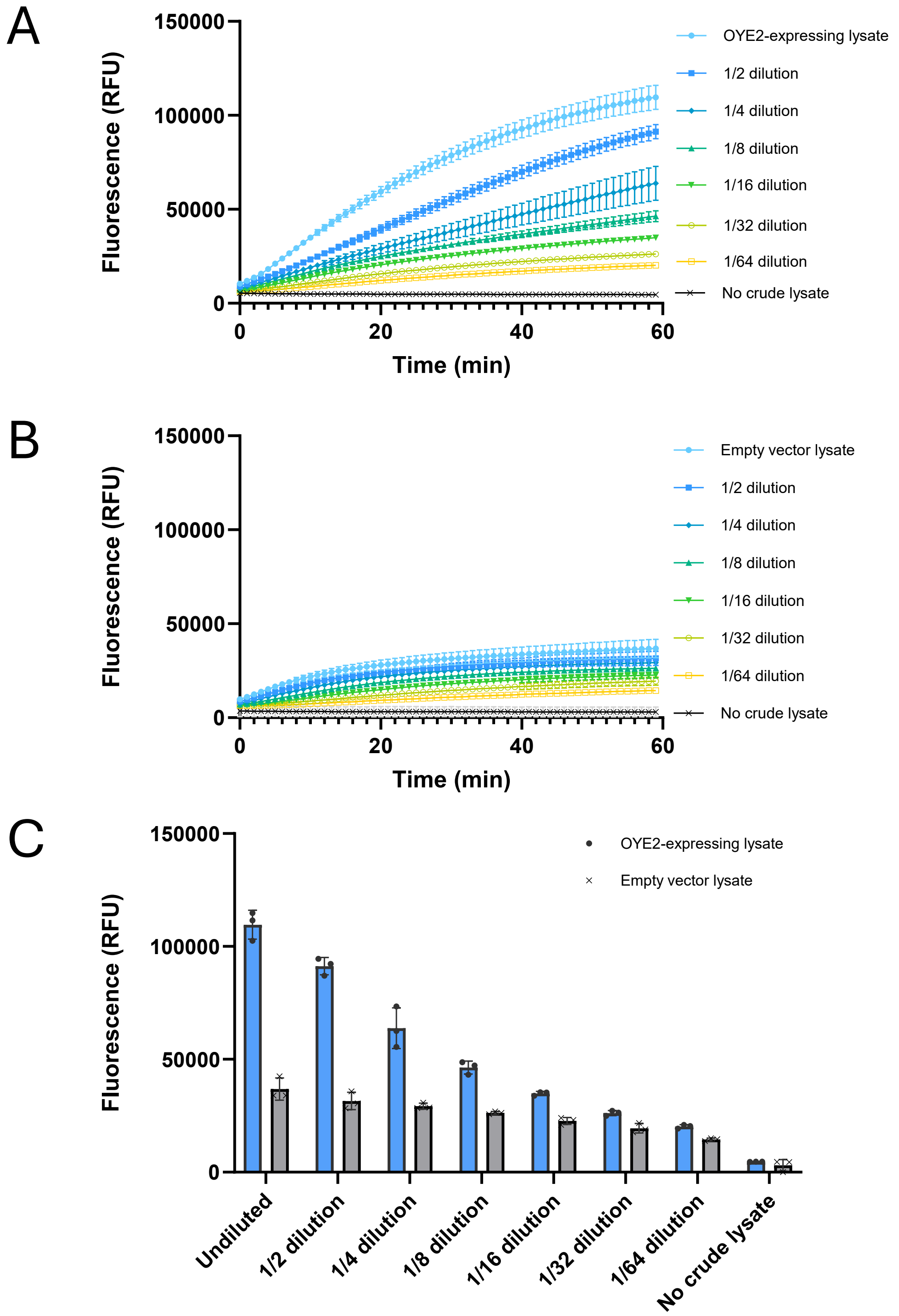

Supplementary Figure S3. Results of fluorescence-based assay by coupled enzyme cascade using crude lysates of *E. coli* cells expressing OYE2 versus those of cells with empty vector. (A) Fluorescence signal from crude lysate of OYE2-expressing cells over time for various dilutions of lysate. (B) Fluorescence signal from the crude lysate of cells containing empty vector over time for various dilutions of lysate. (C) End-point comparisons for OYE2-expressing vs. non-expressing (empty vector) lysates assayed by fluorescence-based coupled enzyme cascade.

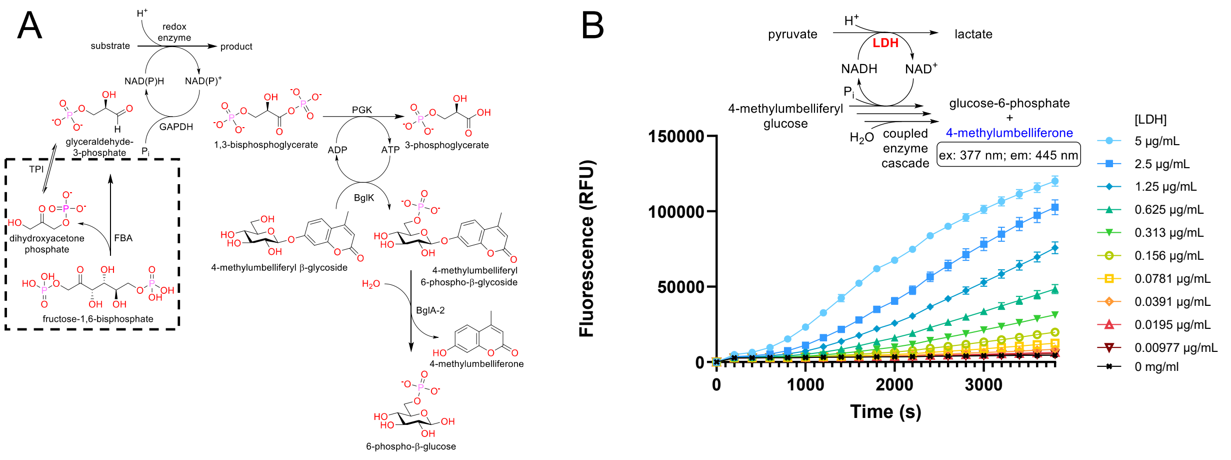

Supplementary Figure S4. (A) extended coupled enzyme cascade including FBA and TPI for *in situ* generation of G3P from FBP. Changes from standard coupled enzyme cascade are indicated by dashed line box. (B) Fluorescence-based assay of the reduction pyruvate catalyzed by LDH (at varied concentration) with an extended coupled enzyme cascade including coupling enzymes (FBA, TPI, GAPDH, PGK, BglK, & BglA-2) and their (co)substrates (FBP, NADH, Pi, ADP, MU-Glc).

Supplementary Table S3. Z' values and signal-to-noise ratio for LDH assays performed by fluorescence-based assay using an extended coupled enzyme cascade including coupling enzymes (FBA, TPI, GAPDH, PGK, BglK, & BglA-2) and their (co)substrates (FBP, NADH, Pi, ADP, MU-Glc).

Concentration of LDH (µg/ml)	Z'	S/N
5	0.91	550
2.5	0.84	500
1.25	0.83	360
0.625	0.78	220
0.313	0.73	140
0.156	0.57	78
0.0781	0.18	40
0.0391	0.32	20
0.0195	-0.025	8.9
0.00977	-0.50	4.9
0.00488	-1.5	2.8
0.00244	-5.4	1.0

Supplementary Table S4. Cost analysis of assay protocols by coupled enzyme cascade

Costs for standard assay by coupled enzyme cascade					
**Components Used**	**Supplier**	**Cost/unit ($CAD)**	**mg/unit**	**mg per rxn**	**Cost ($CAD)**
NADH	Sigma	116	500	0.0035	0.000812
MU-Glc	Sigma	100	100	0.0169	0.0169
G3P	Sigma	407	8	0.00850	0.433
GAPDH (GapA) enzyme	In-house	250	10	0.001	0.025
PGK enzyme	In-house	250	10	0.001	0.025
BglK enzyme	In-house	250	10	0.001	0.025
BglA-2 enzyme	In-house	250	10	0.001	0.025
					0.55

Costs for assay using extended coupled enzyme cascade					
**Components Used**	**Supplier**	**Cost/unit ($CAD)**	**mg/unit**	**mg per rxn**	**Cost ($CAD)**
NADH	Sigma	116	500	0.0035	0.000812
MU-Glc	Sigma	100	100	0.0169	0.0169
F6BP	Sigma	129	1000	0.020303	0.00262
FBA enzyme	In-house	250	10	0.001	0.025
TPI enzyme	In-house	250	10	0.001	0.025
GAPDH (GapA) enzyme	In-house	250	10	0.001	0.025
PGK enzyme	In-house	250	10	0.001	0.025
BglK enzyme	In-house	250	10	0.001	0.025
BglA-2 enzyme	In-house	250	10	0.001	0.025
					0.17

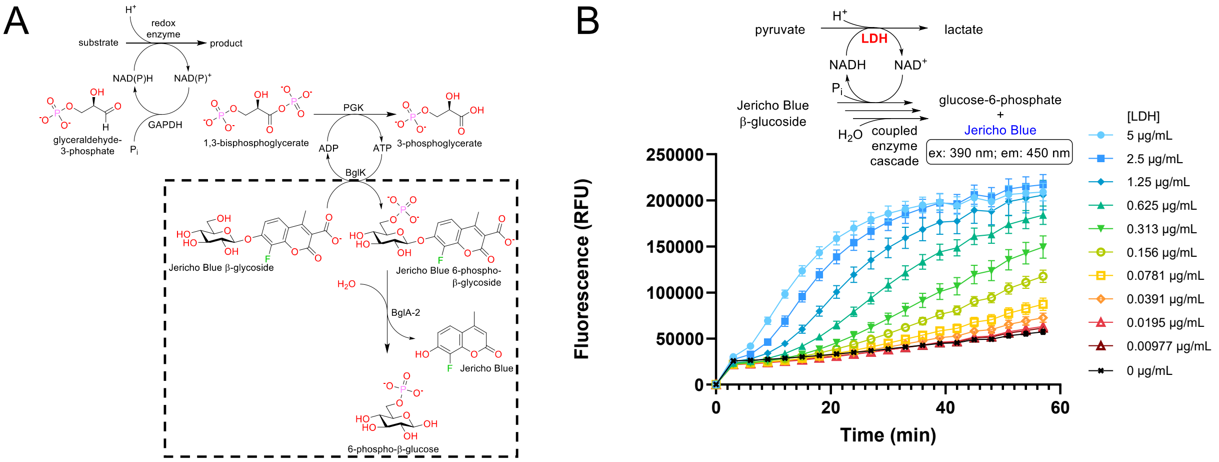

Supplementary Figure S5. (A) modified coupled enzyme cascade using Jericho Blue as a fluorescence reporter. JB-Glc was used in place of MU-Glc and changes from the standard coupled enzyme cascade are indicated by dashed line box. (B) Fluorescence-based assay of the reduction pyruvate catalyzed by LDH (at varied concentration) with a modified coupled enzyme cascade using JB-Glc (instead of MU-Glc).

Supplementary Table S5. Z' values and signal-to-noise ratio for LDH assays performed by fluorescence-based assay with a "Jericho Blue" reporter, using a coupled enzyme cascade including coupling enzymes (GAPDH, PGK, BglK, & BglA-2) and their (co)substrates (FBP, NADH, Pi, ADP, JB-Glc).

Concentration of LDH (μg/ml)	Z'	S/N
5	0.76	65
2.5	0.74	59
1.25	0.62	55
0.625	0.70	47
0.313	0.52	34
0.156	0.54	22
0.0781	0.053	11
0.0391	-0.48	5.7
0.0195	-2.2	2.78
0.00977	-3.5	1.6
0.00488	-20	0.36
0.00244	-1800	0.0035

Supplementary Table S6. Plasmids used for expression of enzymes used in the coupled enzyme cascade(s)

Plasmid	Protein encoded	Source	Addgene
pTrcHis-BglK	BglK	[33]	#252958
pET28-SpBglA-2	BglA-2	This study, based on [34]	#252910
pET28-gapA	GAPDH (*E. coli* GapA)	This study	#252947
pET28-gapA (G188T+P189K)	GapA G188T+P189K	This study	#252948
pET28-pgk	PGK	This study	#252949
pET28-fbaA	FBA (*E. coli* FbaA)	This study	#252950
pET28-tpiA	TPI (*E. coli* TpiA)	This study	#252951
pET28-OYE2	OYE2	This study	#252953

Supplementary Table S7. Primers used for cloning genes encoding enzymes expressed in this study.

Target	Purpose	Sequence (5′ → 3′)
gapA	PCR for cloning (forward primer)	cgcgcggcagccatatgactatcaaagtaggtatcaacgg
	PCR for cloning (reverse primer)	gtcgacggagctcgaattcggatccttatttggagatgtgagcgatcag
	Mutagenesis (G188T+P189K; sense)	cagaaaaccgttgataccaagtctcacaaagactgg
	Mutagenesis (G188T+P189K; anti)	ccagtctttgtgagacttggtatcaacggttttctg
pgk	PCR for cloning (forward primer)	cgcgcggcagccatatgtctgtaattaagatgaccgatctg
	PCR for cloning (reverse primer)	gtcgacggagctcgaattcggatccttacttcttagcgcgctcttc
fbaA	PCR for cloning (forward primer)	cgcgcggcagccatatgtctaagatttttgatttcgtaaaacc
	PCR for cloning (reverse primer)	gtcgacggagctcgaattcggatccttacagaacgtcgatcgcgttc
tpiA	PCR for cloning (forward primer)	cgcgcggcagccatatgggtaactggaaactgaacg
	PCR for cloning (reverse primer)	gtcgacggagctcgaattcggatccttaagcctgtttagccgcttc
OYE2	PCR for cloning (forward primer)	tgccgcgcggcagccatatgccatttgttaaggactttaagc
	PCR for cloning (reverse primer)	cggagctcgaattcggatccttaatttttgtcccaaccgagtttt
